# Characteristics and DGT Based Bioavailability of Cadmium in the Soil–Crop Systems from the East Edge of the Dongting Lake, China

**DOI:** 10.3390/ijerph20010030

**Published:** 2022-12-20

**Authors:** Jun Guo, Zhiying Wei, Chao Zhang, Cong Li, Liangliang Dai, Xin Lu, Kaiqi Xiao, Xiong Mao, Xiuwen Yang, Yiming Jing, Jiaquan Zhang, Wei Chen, Shihua Qi

**Affiliations:** 1 Changsha Natural Resources Comprehensive Investigation Center, China Geological Survey, Changsha 410600, China; 2 Hubei Key Laboratory of Mine Environmental Pollution Control and Remediation, Hubei Polytechnic University, Huangshi 435003, China; 3 School of Environmental Studies and Hubei Key Laboratory of Yangtze Catchment Environmental Aquatic Science, China University of Geosciences, Wuhan 430078, China; 4 School of Environmental Science and Engineering, Hubei Polytechnic University, Huangshi 435003, China; 5 State Key Laboratory of Biogeology and Environmental Geology, China University of Geosciences, Wuhan 430078, China

**Keywords:** cadmium (Cd), diffusive gradients in thin-films (DGT), bioavailability, food safety, Dongting Lake

## Abstract

Contamination of heavy metals (including the cadmium, Cd) in agricultural soils has become an increased issue, posing a threat to the crop safety and human health. In order to evaluate the contamination characteristics and bioavailability of Cd in the soil–crop systems from the East edge of the Dongting Lake, four kinds of agricultural products for typical crops (rice, peanut, sweet potato, and corn) and corresponding rhizosphere soils were collected and analyzed for the Cd concentrations. The technique of diffusive gradients in thin-films (DGT) was applied to evaluate the Cd bioavailability in the rhizosphere soils. Concentrations of Cd ranged from 0.04 to 2.95 mg/kg (average 0.24 mg/kg) with 73.9% sites above the background levels, especially for paddy soils. Cd concentrations in the agricultural products ranged from 0.01 to 2.19 mg/kg (average 0.18 mg/kg), with Cd enrichment observed in the peanut samples. No obvious correlations (R^2^ < 0.25) were observed between the Cd concentrations in the agricultural products and total Cd concentrations in the rhizosphere soils, this indicated that the total Cd concentrations in the soils cannot predict the concentrations in the agricultural products of crops. While the DGT measured Cd concentrations showed good correlations (R^2^ = 0.64–0.90) with the concentrations in the most agricultural products of crops, which may be used to evaluate the safety of the soil and further safety of the agricultural products of crops. Overall, DGT showed a good potential for prediction of heavy metal bioavailability in soil since the DGT technique can simulate the sustained supply of heavy metals from solid to liquid in the soils.

## 1. Introduction

Cadmium (Cd) is one of the toxic heavy metals that can pose a threat to human health [1,2]. Heavy metals are released to the environment from natural processes, as well as from industrial and agricultural activities [3,4]. Nowadays, mining and smelting activities are the major sources of heavy metals emitting to the environment [4,5], through discharging huge amount of wastewater, exhaust gas, and waste residues containing heavy metals. This led to serious metal contaminations in waters [6], surface soils [5,7], and crops [8,9] near the mining and smelting areas, which have been extensively documented around the world [10], with elevated concentrations of heavy metals measured.

Soil is a key part of terrestrial ecosystems and one of the most important resources for human survival and social development [11,12,13]. Soil heavy metal contamination has been a global environmental issue [12,14]. The average concentrations of Cd in uncontaminated soil are 0.06–1.1 mg/kg, while 7% of China’s farmland is at risk of Cd pollution [15,16]. The concentrations of heavy metals in soils are directly related to the quality and safety of agricultural products of crops and the health of human and agricultural ecosystems [14,17,18] because heavy metals in the agricultural soils can transfer to the crops even when there are low concentrations and bioaccumulate through the food chains [19,20,21]. World Health Organization (WHO) set the safety thresholds of Cd as 0.4 mg kg^−1^ for rice, and 0.1 mg kg^−1^ for corn, peanut, and sweet potato [22]. Therefore, it is necessary to study the occurrences and transfer of Cd in the soil–crop systems to ensure the safety of agricultural products of crops and human health.

The transfer of heavy metals from soil to the crops was related to bioavailable species of heavy metals in the soils [23]. Traditionally, the bioavailability of heavy metals in soils/sediments was assessed by specific species from chemical extraction methods using different chemical solutions. However, these methods, which are time-consuming and operationally complex, can only provide chemical species of heavy metals but not the true available species to the crops, and cannot describe the dynamic movements of heavy metals from the soil solids to solutions and then to the crops [24,25]. The technique of diffusion gradient thin-films (DGT), as a passive sampling method for *in-situ* monitoring of heavy metals, can not only reflect the static process shown by traditional chemical extraction methods, but also reflect the dynamic release from soil solids to solutions and then absorption by the crop roots [23,26]. The dynamic process [27], which can simulate the uptake process of heavy metals by the crops, has been widely used to study the environmental behavior of pollutants under laboratory and natural conditions [25,26]. Nolan et al. [23] found that plant Zn and Cd concentrations were highly related to the DGT measurement compared with different methods (total soil concentration extraction, CaCl_2_ extraction, isotope dilution, and Donnan dialysis), and concluded that the kinetic process from the labile solid-phase to solution in the soil measured by DGT played an important role in Zn and Cd uptake by wheat. Tian et al. [25] used DGT and traditional chemical extraction methods to study the bioavailability of heavy metals in the rhizosphere soils of rice, and concluded that DGT measurement can represent bioavailability that was independent from main physical-chemical properties of soils but not traditional chemical extraction methods. Liu et al. [28] applied the DGT to evaluate the bioavailability of heavy metals in the soils near a smelter, showing that the DGT measurement was an effective method to investigate the release–resupply of the labile-HMs. DGT may potentially be a useful tool to evaluate the bioavailability of Cd in the soils and assess the safe application for agricultural soils.

Thus, the purpose of this study is (1) to investigate the occurrences of Cd in agricultural soil and agricultural products for four typical crops from the eastern edge of the Dongting Lake in Hunan, (2) to attempt to apply the DGT technique to assess the Cd bioavailability in soils, and (3) to predict the Cd concentrations in the agricultural products to ensure the security of agricultural products and to further evaluate the safety of the agricultural soils in the study area based on the DGT technique.

## 2. Materials and Methods

### 2.1. Site Description and Sample Collection

The study area is located on the East edge of the Dongting Lake basin in Hunan Province. It is a subtropical continental monsoon climate with four distinct seasons and the landscapes with plains and hills. The annual average temperature is 17 °C, with abundant rainfall and sufficient sunshine. In this area, there is sufficient sunshine and water supply during the growth season for plants, which is suitable for agriculture. According to the previous studies [29,30], the heavy metals in the soils of plain area are greatly affected by the Xiangjiang River in the upper reaches, since the parent materials for these soils originated from the river/lake sediments, but the heavy metals in the Quaternary clay soil from the downland area are less affected due to the higher elevation of the downland area than the plain area; and the heavy metals in the north were originated from the old stratums. The soil types are mainly red soil and paddy soil. The main crops planted in the study area are the rice in the paddy soils, and the corn, peanut, and sweet potato in the dry land soils, respectively.

During the harvest period of crops, a total of 92 pair samples for edible parts of crops (namely agricultural products, AP) and the corresponding rhizosphere soils (RS) were collected in the major agricultural areas from the East edge of the Dongting Lake. Each sample was composed of ca. 10 sub-samples, which accounted for ca. 1.0–2.0 kg of fresh weight for agricultural products and 1.5 kg for soils, respectively.

### 2.2. Soil, Agricultural Products, and DGT Analysis

The rhizosphere soil (RS) samples and agricultural product (AP) samples were pretreated according to the well-documented methods for the Cd detection [9]. The RS was naturally air-dried, grinded, and passed through a 100-mesh sieve. RS samples were then digested with the HCl-HNO_3_-HF-HClO_4_ method. The final volume was adjusted to 10 mL. The AP samples were washed with pure water and then dried at 65 °C to constant weight for pulverizing. The AP samples were digested with the HNO_3_ and H_2_O_2_ method, and the final volume was adjusted to 50 mL. Cd in the RS and AP samples were analyzed and determined by inductively coupled plasma mass spectrometry (ICP-MS, X2, Thermo Fisher Scientific, Germany) under the optimized conditions.

The DGT experiment was conducted according to the previous studies for soil bioavailability [25,26]. The air-dried RS samples were passed through a 10-mesh sieve for DGT deployment. RS samples were wetted to ca. 80% field capacity by ultrapure water. The soil and water were mixed thoroughly using a plastic spatula until a smooth paste was formed. The soil samples were sealed for 48 h for equilibration and aging at room temperature before DGT deployment. DGT devices were then carefully placed in the soil with the exposure area facing the wet soils and making sure there is complete contact between the DGT window and the soil. The temperature was recorded and the DGT devices were retrieved after ca. 24 h. The DGT devices were washed by ultrapure water, and the binding gels were taken out for extracting with 1 mL HNO_3_ (1 mol/L) in the microvials and shaking for 8 h. The extracts were then filtered by a PES disposable filter (0.22 μm). The concentrations of Cd in the elution solution were analyzed by ICP-MS (X2, Thermo Fisher Scientific, Germany) with the Rh as the internal standard.

### 2.3. Geo-Accumulation Index and Bioconcentration Factor

The geo-accumulation index (*I_geo_*) was used as a quantitative index to assess the degree of heavy metal pollution in the soil and to evaluate the impact of soil heavy metal pollution caused by both the natural and anthropogenic effects [31]. It can be calculated by Equation (1):

(1) $$I_{geo}=log_{2}\left[\frac{C}{1.5\times B}\right]$$

where *C* is the measured Cd concentration (mg/kg) in the soil, and *B* is the geochemical background value of Cd in the soil (mg/kg, the soil background value of Cd in Hunan Province is 0.126 mg/kg [32]). When *I_geo_* ≤ 0, it means that the soil is unpolluted; 0 < *I_geo_* ≤ 1, it means the soil is between unpolluted to moderately polluted; 1 < *I_geo_* ≤ 2, it means the soil is moderately polluted; 2 < *I_geo_* ≤ 3, it means the soil is between moderately polluted to strongly polluted; 3 < *I_geo_* ≤ 4, it means the soil is strongly polluted; 4< *I_geo_* ≤ 5, it means the soil is between strongly polluted to extremely polluted; 5 < *I_geo_* ≤ 10, it means the soil is extremely polluted [31].

The bioconcentration factor (BCF) is an index to evaluate the ability of the agricultural products from crops to accumulate the Cd from the rhizosphere soil. The BCF can be calculated as following in Equation (2):

(2) $$\mathrm{BCF}=\frac{C_{\mathrm{crop}}}{C}$$

where *C*_crop_ is the Cd concentration in agricultural products (mg/kg).

### 2.4. Quality Assurance and Quality Control

The quality assurance and quality control procedures were conducted throughout the field sampling, sample pretreatment, and instrument analysis. The purity of all the reagents involved in the experiments was a guaranteed reagent level or above. All the glassware was soaked in a 10% HNO_3_ solution for more than 24 h before use, washed with ultrapure water, and kept in the Teflon or other metal-free containers before use.

During the sampling, the independence and representativeness of the samples were obtained, and the cross-contaminations were avoided during the transport. The procedural blanks, parallel samples, and standard reference materials were prepared during the sample pre-treatment and then analyzed as the unknown samples to avoid the possible contaminations during the experiment, in order to ensure the accuracy of sample determination and verify the accuracy of the method, respectively. The recoveries of Cd were 94.5–103%, and the method detection limit for Cd was 0.002 mg/kg.

## 3. Results and Discussion

### 3.1. Cd Concentrations in Agriculatural Soils

The Cd concentrations in soils ranged from 0.04 to 2.95 mg/kg with an average of 0.24 and the median of 0.18 mg/kg (Table 1), respectively. The coefficients of variations (CV) for the soil were 1.58, indicating that the Cd concentrations in soils changed dramatically. The average concentration of Cd in the soils from this study was similar to the results from the US (0.27 mg/kg) [33] and lower than in New Zealand (0.32 mg/kg) [34] and Zambia (0.53 mg/kg) [35], but higher than in Malaysia (0.12 mg/kg) [36] and Australia (0.13 mg/kg) [37].

**Figure 1:**
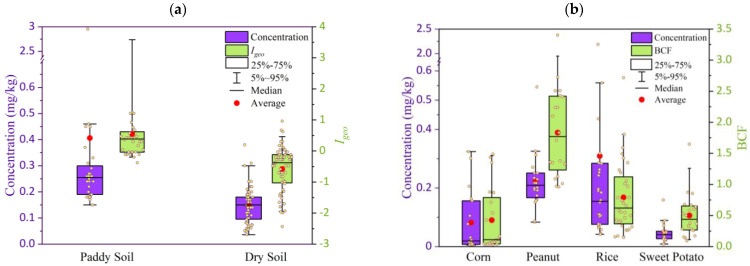
Concentrations and *Igeo* of Cd for soil sampling (**a**) and concentrations and BCF of Cd in agricultural products for crop samples (**b**). (The small yellow points are the concentrations for individual samples).

The concentrations of Cd (Figure 1a) in the paddy soils (average ± SD, 0.41 ± 0.60 mg/kg) were higher than in the dry soils (0.15 ± 0.07 mg/kg); this may be because the higher concernments of organic matter in the paddy soils can adsorb or attract more Cd than in dry soils and the more frequent irrigating in the paddy soil than in the dry soils that input more Cd in the soils [28].

The *I_geo_* values ranged from −2.44 to 3.92 with an average of −0.19 (Table 1), showing that there is not significant contamination in the soil overall. However, the *I_geo_* values were ranged from −0.38 to 3.92 (0.55 ± 0.94) in the paddy soils, which were higher than in dry soil (ranged from −2.44 to 0.96, −0.58 ± 0.74), indicating that the pollution status for the paddy soils was unpolluted to moderately polluted even strongly polluted (*I_geo_* for 3 sites was >3), while the dry soils were not polluted.

### 3.2. Cd Concentrations in Agricultural Products

The Cd concentrations in the agricultural product (AP) samples ranged from 0.01 to 2.19 mg/kg with the average of 0.18 mg/kg (Table 1). The CV for the AP samples were 0.48, which is less than CV for soils, indicating that the change of Cd concentrations in soils was higher than in agricultural products. Among the agricultural products for four crops, the average Cd concentrations (Figure 1b) were the highest in the rice (0.31 ± 0.46 mg/kg), followed by the peanut (0.22 ± 0.10 mg/kg). The average concentrations of Cd in the APs were lower than in East Spain (1.47 ± 2.16 mg/kg) [38], higher than in the US (0.08 mg/kg) [39], and similar to the rice concentrations in Shaanxi (0.22 mg/kg) [40] and Guangxi (0.16 mg/kg) [21] of China.

The overall BCF was in the range of 0.03 to 3.40 with the average of 0.88 (Table 1), showing that there is no significant enrichment. The BCFs for corn and sweet potato were less than 1 (Figure 1b), indicating that these two crops did not enrich Cd from the soil. However, the BCFs were larger than 1 for most peanut samples (0.96 to 3.40, average 1.83) and some rice samples (0.15 to 1.72, average 0.79); this means that the peanuts can easily enrich Cd and rice may enrich Cd in some conditions.

**Figure 2:**
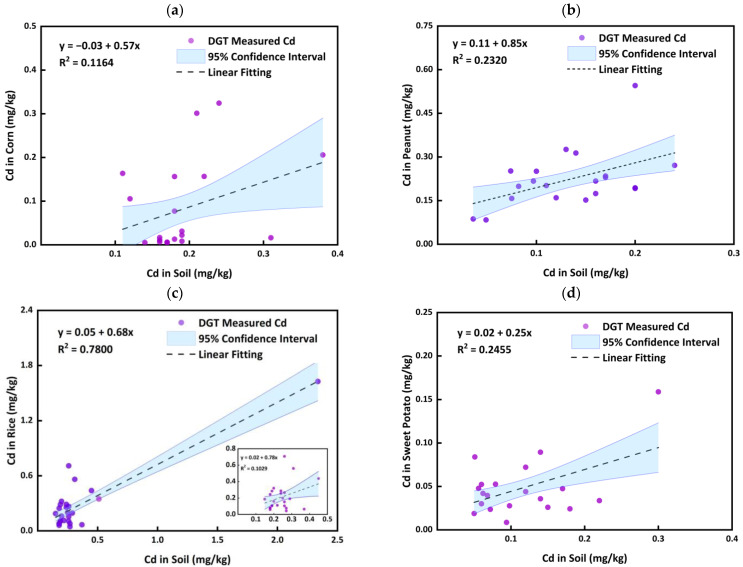
Relationship of Cd concentrations in agricultural product samples for corn (**a**), peanut (**b**), rice (**c**), small figure indicates the relationship which excludes the extremely high concentration, and sweet potato (**d**), and corresponding soils.

The concentration correlations between agricultural product samples and corresponding rhizosphere soils were set up to observe their relationship (Figure 2). For most crops, the correlation coefficients (R^2^) were less than 0.25. The high R^2^ value for rice was quite high (0.7800, Figure 2c) due to an extremely high concentration (>2 mg/kg) in the soil; it then changed to 0.1029 when excluding the extremely high concentration (see the small figure in Figure 2c). This indicates no obvious relationship between the heavy metals in the four agricultural products and the total concentrations of Cd in the soil. A similar result was also found in the previous study for the wheat [23].

Generally, it is believed that the concentrations of heavy metals in agricultural products are closely related to the bioavailable species of heavy metals in the soil. While the bioavailability of Cd in the soil are not directly related to total concentrations of Cd in the soils [41], since many parameters may affect the bioavailable Cd in the soil, such as pH [28], parameter materials [28], DOM [42], and the kinetic release of Cd from soil solids to soil solutions and then the roots [27]. Moreover, the transport of Cd from soil to plants is closely related to the transporters in the root system, resulting in obvious differences in the occurrences of Cd by different crops [43]. Thus, it may be seen that it is very difficult to directly predict the heavy metal concentrations in agricultural products of the crops according to the total concentrations of heavy metals in the soil only (as shown in Figure 2) without any supplementing of other measurements of the soils. We need a useful and practical tool to assess the bioavailability of Cd in the soil and then to predict the Cd concentrations in the agricultural products for the safe application of the agricultural soils and security of agricultural products.

### 3.3. DGT Measurement of Cd

Cd in the soils were measured using the DGT technique, and the results are shown in Table 2 for the soils growing different crops. It can be found that the DGT concentrations for soils growing the rice (1.85 ± 1.88 μg/L) were higher than the other three crops (1.70 ± 0.79 μg/L for the corn, 1.34 ± 1.01 μg/L for the peanut, and 1.19 ± 0.99 μg/L for the sweet potato), which is consistent with the soil concentration of Cd. The CV of DGT concentrations (0.46–0.85) for dry land soils (growing the corn, peanut, and sweet potato) were less than CV (1.01) for paddy soils (growing the rice).

**Figure 3:**
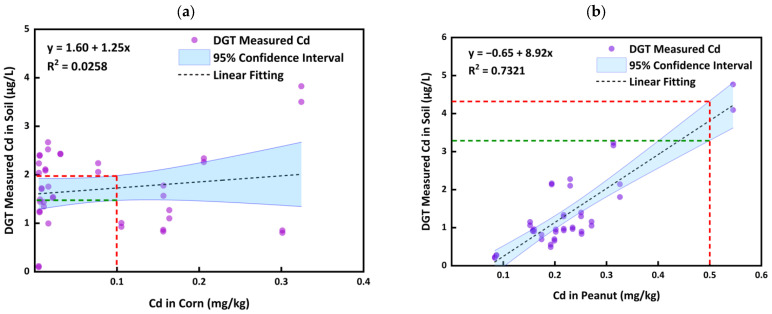

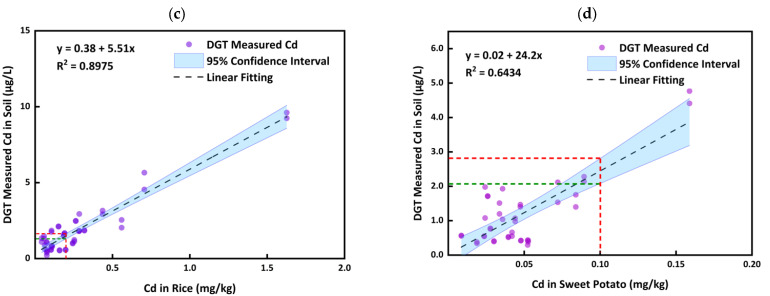
Relationship between DGT measured Cd concentrations and Cd concentrations in the corn, peanut, sweet potato, and rice (The red dashed vertical lines in the figures represent the limit standards for the agricultural products, and the green and red dashed horizontal lines represent the safe and unsafe concentrations predicted by DGT measurement, respectively).

Overall, the good correlations (R^2^ = 0.6434–0.8975) were observed between the DGT measured concentrations of Cd and Cd concentrations in agricultural products (Figure 3), except for the corn. Comparing with Figure 2, it is obvious that DGT can better reflect the concentrations of heavy metals in agricultural products (for the peanut, rice, and sweet potato). This results are in agreement with other studies [42,44]. According to good correlations between DGT measured Cd concentrations in the soil to the Cd concentrations in agricultural products, we may use the DGT technique to link the Cd concentrations in the soils to the agricultural products without the Cd concentrations in the soils extracted by the traditional chemical methods. Thus, it may apply the DGT measured Cd concentrations in the soils to predict the Cd concentrations in the APs, and to further evaluate the safety of agricultural soils, thereby ensuring the security of agricultural products for human health.

### 3.4. Bioavailability of Cd and Safety Assessment of Agricultural Soils

The principle of DGT used for the determination of the availability of Cd in soil is that the dynamic process is very close to the absorption process of Cd in soils by crop roots [41], which can reflect the decrease in Cd concentrations near the roots when the roots absorb Cd in the soil solutions. The release process of Cd from soil solids into soil solutions accurately predicted Cd accumulation in rice grains or direction to the roots (such as peanut or sweet potato).

According to the previous studies, DGT measurement quantitatively incorporates the main factors affecting bioavailability [25], so it may be a good tool to assess the bioavailability of heavy metals. Since good correlations between DGT measured Cd concentrations in soils and the Cd concentrations in the peanut, rice, and sweet potato were observed (Figure 3), we attempt to predict the Cd concentrations agricultural products according to the DGT measured Cd concentrations in the soils, to ensure the security of agricultural products and safe application of agricultural soils on the East edge of the Dongting Lake.

The safety thresholds for Cd in the rice/sweet potato and peanut are 0.1 mg/kg and 0.5 mg/kg, respectively, according to the National Food Safety Standard Limits of Contaminants in Foods (GB2762-2017) in China. Upon the results from Figure 3, we can assess the safety of agricultural soils according to the DGT measured Cd concentrations in soils based on the safety thresholds for the peanut, rice, and sweet potato (the red dashed vertical line in Figure 3). Using the 95% confidence interval (Figure 3), we can believe that, when the DGT measured Cd concentrations in soils are lower than the lower confidence limits (green dashed horizontal line in Figure 3), the soils are safe for the specific crop planting; when the DGT measured Cd concentrations in soils were higher than the upper confidence limits (red dashed horizontal line in Figure 3), the soil would be unsafe for the specific crop planting. The estimated safe and unsafe concentrations by the DGT measurements (Table 3) in soils can be applied to predict the safety of the agricultural soils for specific crop planting on the East edge of the Dongting Lake.

It can be seen from Table 3 that, when the DGT concentrations of Cd in the paddy soils, peanut soils, and sweet potato soils were less than 1.30 μg/L, 3.29 μg/L, and 2.08 μg/L, respectively, the Cd concentrations in the rice, peanuts, and sweet potato would be very likely (95% possibility) to fall in the safety thresholds. On the other hand, when the DGT concentrations of Cd in the paddy soil, peanut soil, and sweet potato were higher than 1.64 μg/L, 4.33 μg/L, and 2.81 μg/L, respectively, the Cd concentrations in the rice, peanuts, and sweet potato would be very likely (95% possibility) to exceed the safety thresholds. In this case, the soils are not suitable for planting rice, peanuts, and sweet potatoes. It is necessary to conduct the remediation for agricultural soils or to intervene in growing certain crops, to ensure that the Cd concentrations in the agricultural products meet the food standard limits.

## 4. Conclusions

The soil Cd in the study area ranged from 0.04 to 2.95 mg/kg (average 0.24 mg/kg), with 73.9% sites being higher than the background values, which are relatively polluted by Cd. The Cd in the agricultural products ranged from 0.01 to 2.19 mg/kg (average of 0.18 mg/kg), and enrichment of Cd was observed in the peanut samples. There is no obvious correlation (R^2^ < 0.25) between Cd concentrations in agricultural products and the total concentrations of Cd in soils. Since the good correlations (R^2^, 0.64–0.90) between DGT measured Cd concentrations and the concentrations in the rice, sweet potatoes, and peanuts, DGT techniques are used to evaluate the safety of the soils and the planted crops for agricultural products: when the DGT concentrations of Cd in the paddy soils, peanut soils, and sweet potato soils from the East edge of the Donting Lake were less than 1.30 μg/L, 3.29 μg/L, and 2.08 μg/L, respectively, the Cd concentrations in the rice, peanuts, and sweet potatoes would be under the standard limits. Overall, DGT showed a good potential for the prediction of heavy metal bioavailability in soils to further ensure security of agricultural products and the safe application of the agricultural soils. The microcosmic mechanism should be studied for better understanding the interface process and transfer of heavy metals among soil solids, soil solutions, and crop roots; and more similar studies should be conducted for further verifying and implementing this technique and its application.

## Figures and Tables

**Table 1:** Statistics description for Cd in soil and crop samples.

	Max	Min	Average	Median	SD	CV
Soil (mg/kg)	2.95	0.04	0.24	0.18	0.38	1.58
*I_geo_*	3.92	−2.44	−0.19	−0.12	0.96	−5.10
Crop (mg/kg)	2.19	0.01	0.18	0.12	0.30	0.48
BCF	3.40	0.03	0.88	0.66	0.76	0.66

**Table 2:** DGT measured concentrations of Cd (μg/L) for soils growing different crops.

	Corn	Peanut	Rice	Sweet Potato
Max	3.83	4.77	9.61	4.77
Min	0.09	0.20	0.21	0.29
Average	1.70	1.34	1.85	1.19
SD	0.79	1.01	1.88	0.99
CV	0.46	0.75	1.01	0.84

**Table 3:** DGT predicted safe and unsafe concentrations (μg/L) for different agricultural products calculated according to Figure 3 with a 95% confident interval.

	Peanut	Rice	Sweet Potato
Safe	<3.29	<1.30	<2.08
Unsafe	>4.33	>1.64	>2.81

## Data Availability

The data that support the findings of this study are available from the corresponding author, Wei Chen (wei.chen@cug.edu.cn), upon reasonable request.
